# Supplementary material for: Vasculature segmentation in 3D hierarchical phase-contrast tomography images of human kidneys
Source: bioRxiv. 2024 Aug 26:2024.08.25.609595. Preprint. [Version 1] doi: 10.1101/2024.08.25.609595 (PMC11383006; doi:10.1101/2024.08.25.609595)
Supplement: 1 [file NIHPP2024.08.25.609595V1-supplement-1.pdf]

bioRxiv preprint doi: https://doi.org/10.1101/2024.08.25.609595; this version posted August 26, 2024. The copyright holder for this preprint (which was not certified by peer review) is the author/funder, who has granted bioRxiv a license to display the preprint in perpetuity. It is made available under aCC-BY 4.0 International license.

Supplementary Notes

Supplementary Note 1: Team 1 solution details

Summary: Team 1 proposed a customized U-Net[1] based architecture model using a lightweight encoder named ConvNeXt-tiny[2] with additional stem blocks[3] to improve the feature extraction. A data augmentation technique, including random 3d slice rotation, was acquired to tackle the challenges related to the data. To make sure that the proposed model is optimized well, they trained the model with an ensemble loss combining focal loss[4], dice loss[5], boundary loss[6] and a customized marching cube loss. The main goal of marching cube loss is to directly optimize the surface dice. The data were processed across three axes with large inference size and test time augmentation for inference.

Data Preparation: They trained the proposed model using all available data, including high-resolution and sparsely labeled data. Then, all slices were randomly resized to size 1536*1536. To increase the size of the training data, they sliced the whole volume along different axes and views. Heavy data augmentation, including flipping, scaling, adding noise, and intensity modifications, was utilized. Moreover, they randomly rotated the sampled slice in 3D space as online data augmentation.

Model: The general architecture of the model includes a 2.5D U-Net[1] based architecture stacking 3 consecutive slices on channel dimension as input for the model to leverage 3D information. The encoder part of the model includes the ConvNeXt-tiny model[2] combined with an extra stem block[3] to extract high-resolution features. The decoder part of the model is a standard U-Net decoder. For the loss function, this team utilized a combination of four losses including focal loss[4], dice loss[5], boundary loss[6], and a newly proposed customized Marching Cube loss.

Inspired by surface dice computation, the customized Marching Cube loss replaces the surface area expectation with the ground truth surface area. Therefore, the Marching Cube Loss (MCL) can be calculated as follows:

$$MCL = 1 - \frac{2\sum_i p_i y_i w_i}{\sum_i p_i w_i + \sum_i y_i w_i}$$

In practice, the surface area weight has minimal influence on the final performance of the mentioned loss. So, the loss mentioned above only considers the surface cubes (ground truth foreground and background cube's surface areas are zero). Therefore, ignoring the surface area weight, this team used the mean of foreground, background and surface dice losses as the customized Marching Cube loss, which can be seen below.

$$MCL = 1 - \frac{1}{|K|}\sum_{k \epsilon K} \frac{2\sum_i p_i^k g_i^k}{\sum_i p_i^k + \sum_i g_i^k} \qquad K$$

$$= \{foreground, background, surface\}$$

bioRxiv preprint doi: https://doi.org/10.1101/2024.08.25.609595; this version posted August 26, 2024. The copyright holder for this preprint (which was not certified by peer review) is the author/funder, who has granted bioRxiv a license to display the preprint in perpetuity. It is made available under aCC-BY 4.0 International license.

Training and Inference Details: This team trained the proposed model using a 32 batch size and a gradient accumulation process[7]. AdamW[8] with additional WarmUpConsineAnnealing[9] was used to optimize the model. The model was trained for 30 epochs.
In the testing process, all the slices were converted and resized to 3072*3072. The torch compile algorithm[7] was utilized to accelerate the inference process. Moreover, the data were processed across three axes with large inference sizes and test time augmentation.

Supplementary Note 2: Team 2 solution details

Summary: Team 2 used a customized 3D U-Net[10] model for segmenting the kidney vessels. To overcome the generalizability problem, data augmentation techniques, including random positions and rotations, were used. Eventually, morphological post-processing techniques were used to remove small false positives and improve the model performance.

Model: The general architecture of the proposed neural network is based on 3D U-Net[10]. However, to overcome the problem of gradient vanishing, simple convolution layers were replaced by ResNet blocks[3] in the encoder part of the model. To decrease the computational cost, all traditional up-convolutional layers have been replaced by up-sampling operations.
Online data augmentation techniques, specifically affine transformations such as rotation, scaling, and shearing, were employed to prevent overfitting. This process yielded a substantial increase in the number of patches, reaching over 7k for every epoch.

For the post-processing, the depth-first search algorithm[11] was used to identify the unconnected, distantly placed chunks and remove them to increase the model's overall performance.

Training and Inference Details: This team trained the proposed model with a batch size of 4 and a size of 128*128*32. For optimization, they used Focal loss[4] and cosine decay scheduling[9]. The training lasted two weeks, and the maximum number of epochs was 700. 0.25 was used as the ultimate threshold for the inference to extract binary masks for vessels.

bioRxiv preprint doi: https://doi.org/10.1101/2024.08.25.609595; this version posted August 26, 2024. The copyright holder for this preprint (which was not certified by peer review) is the author/funder, who has granted bioRxiv a license to display the preprint in perpetuity. It is made available under aCC-BY 4.0 International license.

Supplementary Note 3: Team 3 solution details

Summary: Team 3 employed a multifaceted approach to tackle the competition. They used a two-step process to refine sparse labels using dense labels from part of the training dataset. Initially, they trained two different UNet architectures (maxViT512[12] and EfficientNetv2[13]) on densely labeled images to generate supplemental labels for sparsely labeled ones. These models were then further trained using the enhanced datasets, which included both dense and newly generated pseudo labels. The team trained three Unet models: EfficientNetv2, SeResNext101[14], and MaxViT512, and one UNet++[15] using all real labels from all kidneys plus pseudo labels.

Model: The main model, MaxVit512, represents a sophisticated UNet-based architecture combining CNN and Transformer methodologies, tailored specifically for medical image segmentation. It utilizes a hybrid multi-axis vision transformer mechanism, where both convolutional and self-attention mechanisms are employed at each stage of the decoder. This approach significantly enhances the model's ability to distinguish between target objects and the background, crucial for effective segmentation. The MaxVit512 architecture, with its strategic use of a hybrid decoder, ensures high efficiency in segmentation with a reasonable computational and memory footprint. The models typically operate with a batch size of 32, allowing efficient processing of large datasets without compromising performance.

Training and Inference Details: Recognizing the variation in magnification between training and test sets (50µm/voxel for training and public test sets, and 63µm/voxel for the private test set), the team adjusted their training strategy to simulate the lower resolution of the private test set by scaling images down. They applied a ShiftScaleRotate augmentation, which randomly shifts, scales, and rotates the images to enhance model robustness to variations in image presentation. Training and inference were performed along different axes (x, y, and z) to manage varied resolutions and sizes, using solely 2D models. They maintained a consistent model resolution, opting for 512px for most tasks but switching to higher resolutions when supported by the dataset to minimize potential accuracy loss in smaller resolution data.

The team trained models on all available data once they observed stable convergence, maximizing learning from limited datasets. They used dynamic threshold values to maintain stability in model predictions, crucial for consistent segmentation performance. Given the intensity variations across different datasets, they implemented heavy intensity augmentation strategies to enhance model robustness. For final submissions, the team used both a single model and an ensemble of the four models mentioned, with both approaches yielding the same score of 0.727. Their script utilized CUDA for GPU acceleration, ensuring quick prediction processing. Through these strategic maneuvers, the team effectively managed dataset variabilities and optimized their models for high performance. Overall, MaxVit512 scored 0.727, and the ensemble submission also scored 0.727.

bioRxiv preprint doi: https://doi.org/10.1101/2024.08.25.609595; this version posted August 26, 2024. The copyright holder for this preprint (which was not certified by peer review) is the author/funder, who has granted bioRxiv a license to display the preprint in perpetuity. It is made available under aCC-BY 4.0 International license.

Supplementary Note 4: Team 4 solution details

Summary: The fourth team's final model strategy combined 2D and 3D models, utilizing d4 test-time augmentation (TTA)[16] to improve segmentation results. They applied a multi-view TTA approach to the 2D models and conducted training using a 2-fold setup, selecting kidney_2 and kidney_3_dense as validation sets. They ensembled the models by assigning equal weights to both 2D and 3D models. Their training datasets included kidney_1_dense, kidney_2, kidney_3_dense, kidney_3_sparse, and pseudo labels. The team transitioned from slice-wise normalization to stack-wise normalization using percentiles to optimize model learning.

Model: The team's main models included EfficientNet family models with a UnetPlusPlus decoder and SCSE attention[17], integrated from the segmentation_models_pytorch library. EfficientNet-B5 was particularly effective for feature extraction, capturing complex patterns in images. UnetPlusPlus enhanced feature propagation, crucial for accurately delineating boundaries and maintaining spatial details during encoding and decoding. Despite testing various architectures, the EfficientNet-B5-UNet++ model demonstrated superior performance. Their 3D model was based on the NN-Unet architecture but used the DynUnet from the MONAI library, with training over 500 epochs using SGD and a cosine annealing learning rate schedule.

Training and Inference Details: Training involved a multiview setup, stacking images in tensors and slicing along different axes. They employed a weighted sampling approach based on sample sparsity, with denser samples given a standard weight and sparser samples adjusted accordingly. For example, kidney_1_dense had a weight of 1, while kidney_2 was assigned a weight of 0.65. Dynamic augmentations like CutMix[18] (applied with a 0.5 probability), shifts, flips, and brightness adjustments were crucial for model robustness. The 3D model's augmentation strategy was simpler, involving d4 augmentations and random crops, with cropping adjusted to 192x192x192 to focus on volumetric capture.

Pseudo labeling was done using an ensemble of 2D models with data from https://human-organ-atlas.esrf.eu/, excluding some data to prevent leakage. Post-processing techniques, such as multiplying 3D model predictions with 2D model-derived ROI masks, significantly enhanced predictions. Ensembling predictions from 2D and 3D models with equal weighting further boosted performance. The team found that incorporating BoundaryDOULoss[19] early in the competition significantly improved model performance, with a +2% increase in cross-validation metrics, +1.5% on the public leaderboard, and +5% on the private leaderboard.

Supplementary Note 5: Team 5 solution details

Summary: Team 5 solution applied only a 2D segmentation model using HiP-CT slices as inputs. The final predictions were given by ensembling three 2D U-Net models trained on xy, yz and xz axes, respectively. The team trained the model on kidney_1, then kidney_2 and validated on kidney_3. To achieve better results, they applied several technical implementations such as pseudo-labeling from additional data, boundary loss and spatial resolution interpolation.

bioRxiv preprint doi: https://doi.org/10.1101/2024.08.25.609595; this version posted August 26, 2024. The copyright holder for this preprint (which was not certified by peer review) is the author/funder, who has granted bioRxiv a license to display the preprint in perpetuity. It is made available under aCC-BY 4.0 International license.

Pseudo-labelling: Considering that data is important to train an effective deep neural network, the team involved two additional datasets: LADAF-2020-31 kidney[20] and LADAF-2020-27 spleen[21], from the Human Organ Atlas (https://human-organ-atlas.esrf.eu/) into the training. A 4-step training scheme, was applied to gradually generate pseudo labels for the new datasets and incorporate them into training:

1) The team trained a base model on kidney_1 and pseudo-labeled, kidney_2;
2) Kidney_2 was incorporated into the training data together with kidney_1 to re-train the model. Then, they used this model to pseudo-labeled the LADAF-2020-31 kidney.
3) Similarly, they pseudo-labeled LADAF-2020-27 spleen dataset from the model re-trained on kidney_1, kidney_2 and LADAF-2020-31 kidney.
4) Finally, the model was trained on four datasets.

The 4-step training scheme enabled generating pseudo-labels for the new dataset. However, pseudo-labels can introduce training noise with false positives which are difficult to completely remove in this scheme without post-processing. To overcome this problem, the team used soft labels, the probabilities after sigmoid function, as pseudo-labels instead of hard labels.

Loss: As surface dice is one of the metrics in this competition, evaluating the predicted boundaries of the vasculature, the team applied a boundary weighted binary cross-entropy loss as equation:

$$L = -\sum_{i=0}^{i=n-1} (1 + \alpha bound_i)[y_i \, log p(y_i) - (1 - y_i) log(1 - p(y_i))] \ ,$$
$$bound_i = 0 \ if \ y_i \ is \ not \ on \ boundary, 1 \ if \ y_i \ is \ on \ the \ boundary$$

where n is the size of a mini-batch. α is a boundary weight and $bound_i$ is a binary value, indicating if the value y_i is on the boundary. The last term is a typical binary cross-entropy function. The team set α as 0.9 to roughly double the loss on the boundary pixels. Apart from a boundary weighted binary cross-entropy loss, the overall loss function added dice loss[5] and focal loss[4].

Models: Four different 2D backbone networks: effnet_v2_s, effnet_v2_m, maxvit_base and dpn68 were ensembled for the final submission. They are implemented by a python package - Segmentation Models PyTorch (SMP)[22]. The input was cropped from xy, xz and yz axes in size of 512^2, followed by pre-processing methods, such as 2D rotation, intensity contrast changes, horizontally and vertically flipped. After training, the inference was done with a sliding window method with an overlap size of half the input size.

Inference: Team 5 solution highlighted that 3D interpolation to make the voxel sizes of the training data and test data the same is important when performing vasculature segmentation inference on HiP-CT data. In this competition, the training data of kidney 1 to kidney 3 are ~50 µm/voxel, however, the test dataset of kidney 6 is ~63 µm/voxel. Team 5 applied a rescaling operation through 3 axes even though training was performed on 2D slices. The rescaling was implemented by trilinear interpolation and the rescaled data resulted in better performance.

bioRxiv preprint doi: https://doi.org/10.1101/2024.08.25.609595; this version posted August 26, 2024. The copyright holder for this preprint (which was not certified by peer review) is the author/funder, who has granted bioRxiv a license to display the preprint in perpetuity. It is made available under aCC-BY 4.0 International license.

Supplementary Note 6: Useful strategies from other teams

Pre-processing

The kidneys were imaged by HiP-CT which requires the X-ray beam and scan setup to be independently tuned for every individual sample. Thus, scan parameters and hence the ranges of voxel intensities vary often dramatically between samples. This effect introduces weight bias and shifts when training deep neural networks. Therefore, several approaches were applied in this competition to construct a uniform input intensity space for stable training. Popular pre-processing techniques were normalization with global maximum and minimum across three training kidneys and intensity augmentation in training and validation. However, for 2D models, normalization of individual slices performs better than normalization with global maximum and minimum from 3D volume (https://www.kaggle.com/competitions/blood-vessel-segmentation/discussion/469022).

Model Selection

HiP-CT can image intact human organs, with isotropic voxels enabling clear visualization of 3D structures in any orientation. This inherently inspires the application of 3D as well as 2.5D and 2D segmentation models. Therefore, many teams explored different models for this task, reporting that 2D models were more efficient for achieving better results, due to the challenges of setting the hyper-parameters for 3D models. Due to limitations in computational resources for many competitors, 3D model input patches were smaller (input dimensions = $64 \times 64 \times 64$) and thus contained less spatial context, compared to popular input settings of 512×512 in 2D and 2.5D models. If the input dimension were increased in 3D models, the batch size had to be reduced.

Post-processing

For HiP-CT scanning, organs are physically stabilized in cylindrical jars using an ethanol–agar or formalin-agar gel, so the images also contain the irrelevant background areas of the surrounding ethanol and agar. To remove the false positives on those areas, most of the teams created the mask for the kidney using different techniques such as Canny filters from OpenCV package and intensity thresholding. Since connectivity is one of the important factors for vascular trees and models failed in preserving these contextual long dependencies, connected component 3D (cc3d)[23] was popularly used to improve the connectivity of the predictions as a post-processing method.

Supplementary References

1. Ronneberger, O., Fischer, P. & Brox, T. U-Net: Convolutional Networks for Biomedical Image Segmentation. in *Medical Image Computing and Computer-Assisted Intervention – MICCAI 2015* (eds. Navab, N., Hornegger, J., Wells, W. M. & Frangi, A. F.) 234–241 (Springer International Publishing, Cham, 2015). doi:10.1007/978-3-319-24574-4_28.

bioRxiv preprint doi: https://doi.org/10.1101/2024.08.25.609595; this version posted August 26, 2024. The copyright holder for this preprint (which was not certified by peer review) is the author/funder, who has granted bioRxiv a license to display the preprint in perpetuity. It is made available under aCC-BY 4.0 International license.

2. Liu, Z. *et al.* A ConvNet for the 2020s. Preprint at https://doi.org/10.48550/arXiv.2201.03545 (2022).
3. He, K., Zhang, X., Ren, S. & Sun, J. Deep Residual Learning for Image Recognition. in *2016 IEEE Conference on Computer Vision and Pattern Recognition (CVPR)* 770–778 (2016). doi:10.1109/CVPR.2016.90.
4. Lin, T.-Y., Goyal, P., Girshick, R., He, K. & Dollar, P. Focal Loss for Dense Object Detection. in 2980–2988 (2017).
5. Milletari, F., Navab, N. & Ahmadi, S.-A. V-Net: Fully Convolutional Neural Networks for Volumetric Medical Image Segmentation. in *2016 Fourth International Conference on 3D Vision (3DV)* 565–571 (2016). doi:10.1109/3DV.2016.79.
6. Kervadec, H. *et al.* Boundary loss for highly unbalanced segmentation. *Med. Image Anal.* **67**, 101851 (2021).
7. Paszke, A. *et al.* PyTorch: An Imperative Style, High-Performance Deep Learning Library. in *Advances in Neural Information Processing Systems* vol. 32 (Curran Associates, Inc., 2019).
8. Kingma, D. P. & Ba, J. Adam: A Method for Stochastic Optimization. Preprint at https://doi.org/10.48550/arXiv.1412.6980 (2017).
9. Loshchilov, I. & Hutter, F. SGDR: Stochastic Gradient Descent with Warm Restarts. Preprint at https://doi.org/10.48550/arXiv.1608.03983 (2017).
10. Çiçek, Ö., Abdulkadir, A., Lienkamp, S. S., Brox, T. & Ronneberger, O. 3D U-Net: Learning Dense Volumetric Segmentation from Sparse Annotation. in *Medical Image Computing and Computer-Assisted Intervention – MICCAI 2016* (eds. Ourselin, S., Joskowicz, L., Sabuncu, M. R., Unal, G. & Wells, W.) 424–432 (Springer International Publishing, Cham, 2016). doi:10.1007/978-3-319-46723-8_49.
11. Tarjan, R. Depth-first search and linear graph algorithms. in *12th Annual Symposium on Switching and Automata Theory (swat 1971)* 114–121 (1971). doi:10.1109/SWAT.1971.10.

bioRxiv preprint doi: https://doi.org/10.1101/2024.08.25.609595; this version posted August 26, 2024. The copyright holder for this preprint (which was not certified by peer review) is the author/funder, who has granted bioRxiv a license to display the preprint in perpetuity. It is made available under aCC-BY 4.0 International license.

12. Tu, Z. *et al.* MaxViT: Multi-axis Vision Transformer. in *Computer Vision – ECCV 2022* (eds. Avidan, S., Brostow, G., Cissé, M., Farinella, G. M. & Hassner, T.) 459–479 (Springer Nature Switzerland, Cham, 2022). doi:10.1007/978-3-031-20053-3_27.
13. Tan, M. & Le, Q. V. EfficientNetV2: Smaller Models and Faster Training. Preprint at https://doi.org/10.48550/arXiv.2104.00298 (2021).
14. Hu, J., Shen, L. & Sun, G. Squeeze-and-Excitation Networks. in 7132–7141 (2018).
15. Zhou, Z., Rahman Siddiquee, M. M., Tajbakhsh, N. & Liang, J. UNet++: A Nested U-Net Architecture for Medical Image Segmentation. in *Deep Learning in Medical Image Analysis and Multimodal Learning for Clinical Decision Support* (eds. Stoyanov, D. et al.) 3–11 (Springer International Publishing, Cham, 2018). doi:10.1007/978-3-030-00889-5_1.
16. Wang, G. *et al.* Aleatoric uncertainty estimation with test-time augmentation for medical image segmentation with convolutional neural networks. *Neurocomputing* **338**, 34–45 (2019).
17. Roy, A. G., Navab, N. & Wachinger, C. Concurrent Spatial and Channel Squeeze & Excitation in Fully Convolutional Networks. Preprint at https://doi.org/10.48550/arXiv.1803.02579 (2018).
18. Yun, S. *et al.* CutMix: Regularization Strategy to Train Strong Classifiers With Localizable Features. in 6023–6032 (2019).
19. Sun, F., Luo, Z. & Li, S. Boundary Difference Over Union Loss For Medical Image Segmentation. Preprint at https://doi.org/10.48550/arXiv.2308.00220 (2023).
20. Tafforeau, P. *et al.* Complete kidney from the body donor LADAF-2020-31. European Synchrotron Radiation Facility https://doi.org/10.15151/ESRF-DC-572253769 (2021).
21. Tafforeau, P. *et al.* Complete spleen from the body donor LADAF-2020-27. European Synchrotron Radiation Facility https://doi.org/10.15151/ESRF-DC-572244468 (2021).
22. Iakubovskii, P. Segmentation Models Pytorch. *GitHub repository* (2019).
23. Silversmith, W. seung-lab/connected-components-3d: Zenodo Release v1. Zenodo https://doi.org/10.5281/zenodo.5535251 (2021).

bioRxiv preprint doi: https://doi.org/10.1101/2024.08.25.609595; this version posted August 26, 2024. The copyright holder for this preprint (which was not certified by peer review) is the author/funder, who has granted bioRxiv a license to display the preprint in perpetuity. It is made available under aCC-BY 4.0 International license.

Supplementary Figures

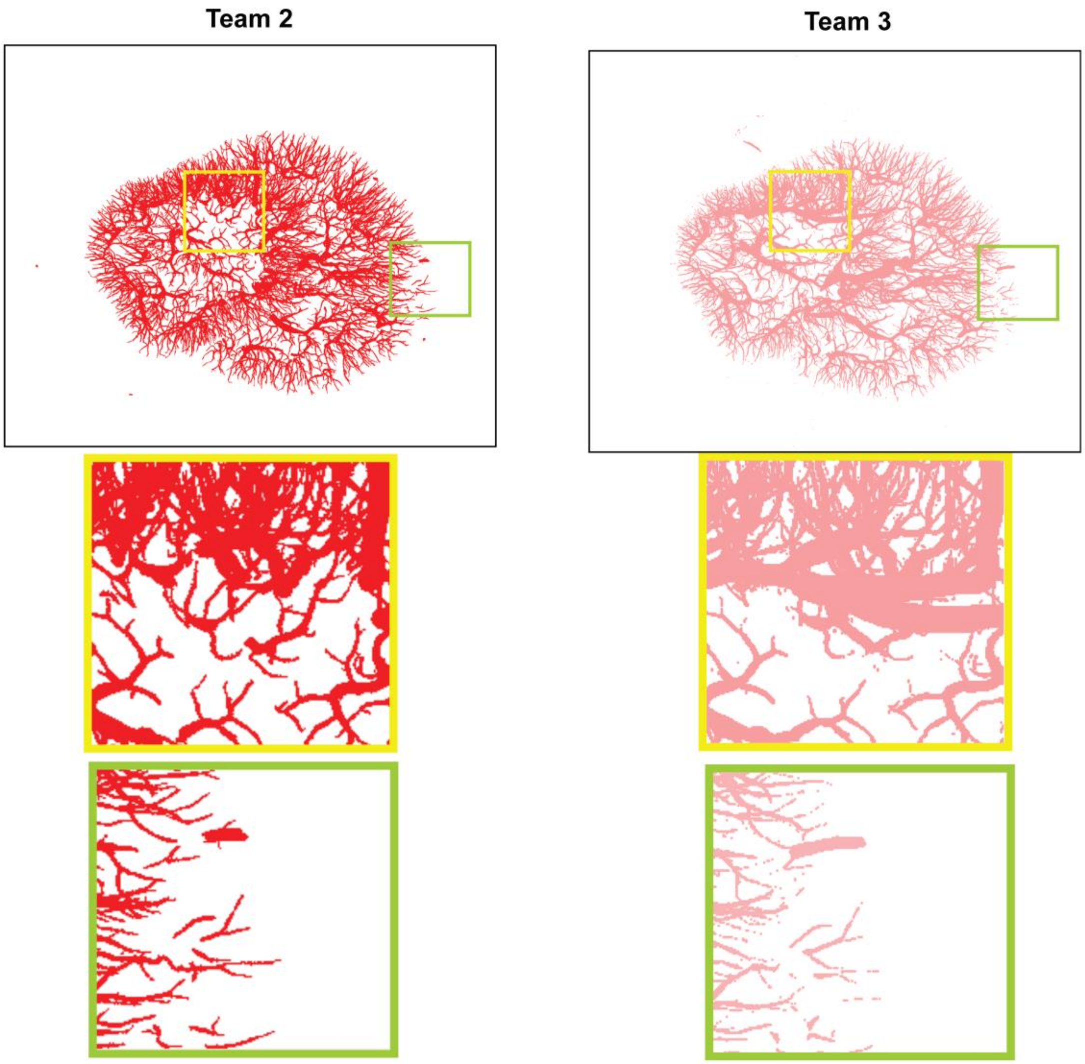

Supplementary Figure 1. Maximum intensity projections (MIP) for Teams 2 and 3, with two insets per team in the yellow and green squares, respectively.

bioRxiv preprint doi: https://doi.org/10.1101/2024.08.25.609595; this version posted August 26, 2024. The copyright holder for this preprint (which was not certified by peer review) is the author/funder, who has granted bioRxiv a license to display the preprint in perpetuity. It is made available under aCC-BY 4.0 International license.

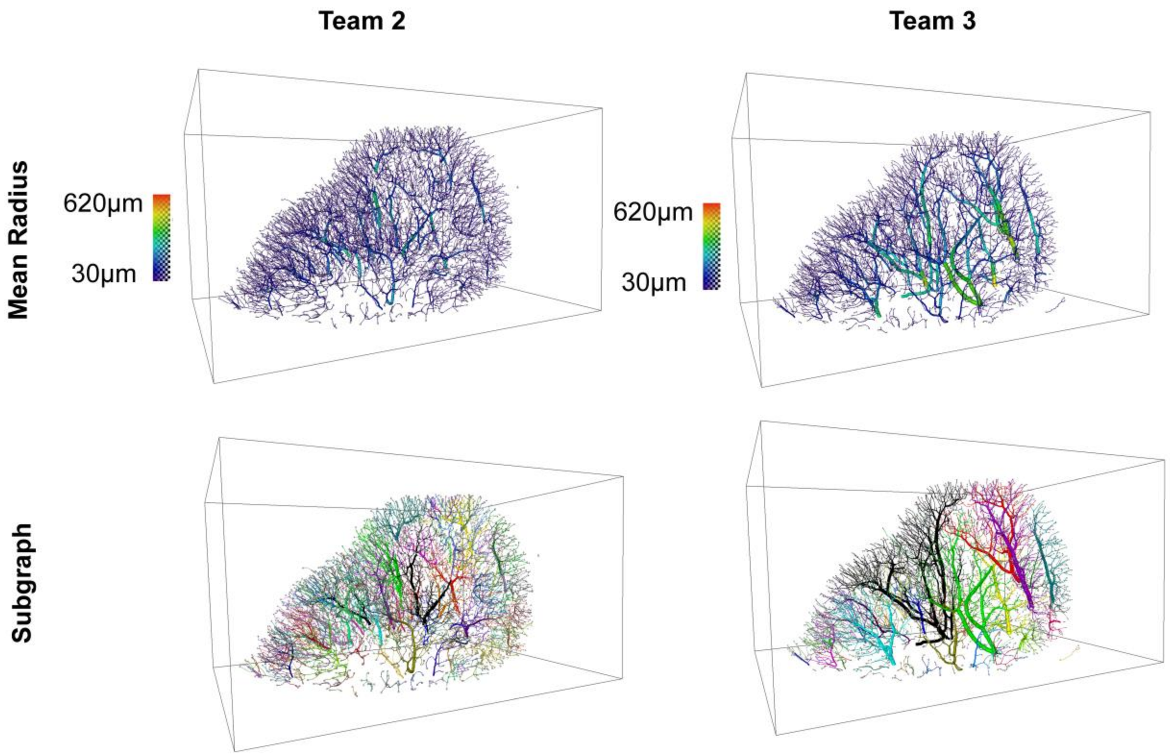

Supplementary Figure 2. The figure shows the skeleton forms of the vessel network for Teams 2 and 3. In each case the vessel network size is proportional to the mean radius in the first row; the color shows the mean radius for each case. The second row is colored with each unconnected subgraph in a different color.

bioRxiv preprint doi: https://doi.org/10.1101/2024.08.25.609595; this version posted August 26, 2024. The copyright holder for this preprint (which was not certified by peer review) is the author/funder, who has granted bioRxiv a license to display the preprint in perpetuity. It is made available under aCC-BY 4.0 International license.

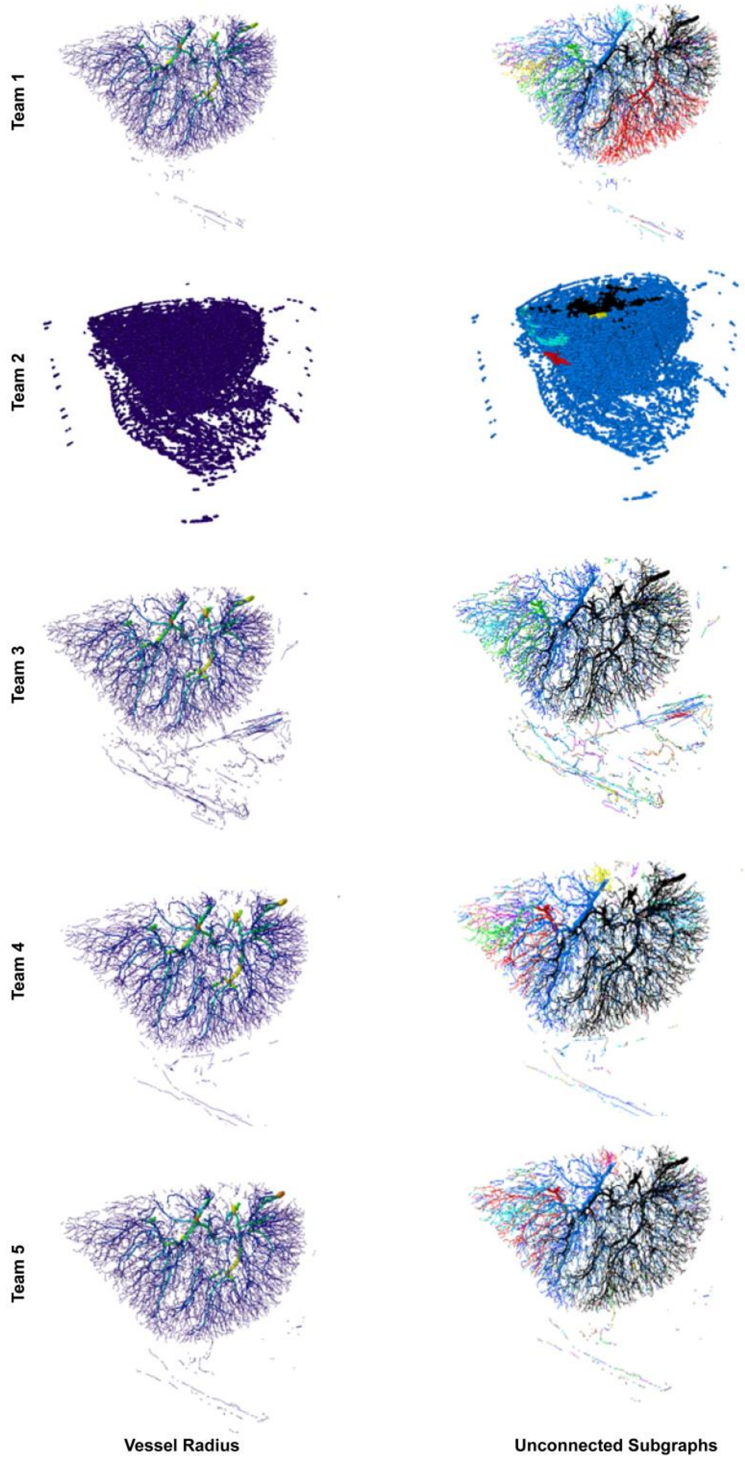

Supplementary Figure 3. Showing the skeleton forms of the vessel network for all teams for the public test data. In each case the vessel network size is proportional to the mean radius in the first column; the color shows the mean radius for each case. The second column is colored with each unconnected subgraph in a different color.

bioRxiv preprint doi: https://doi.org/10.1101/2024.08.25.609595; this version posted August 26, 2024. The copyright holder for this preprint (which was not certified by peer review) is the author/funder, who has granted bioRxiv a license to display the preprint in perpetuity. It is made available under aCC-BY 4.0 International license.

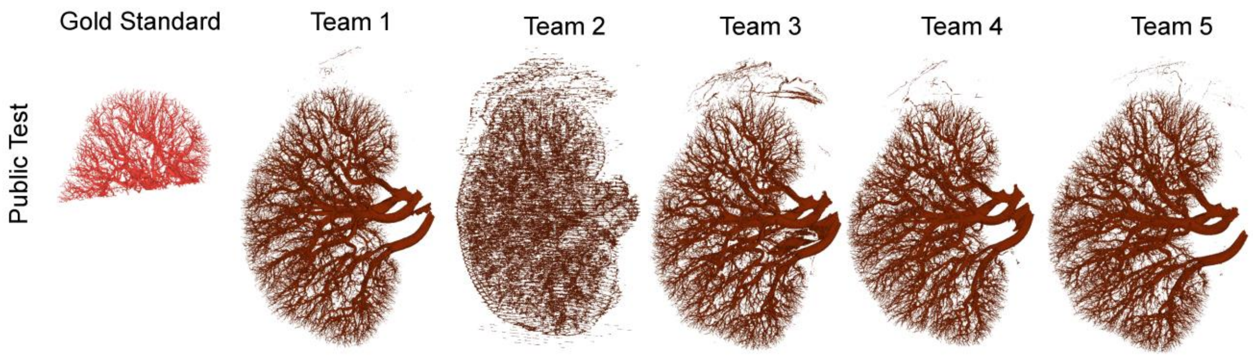

Supplementary Figure 4. Visualization of the 3D output for inference of each team on the whole intact kidney datasets for the public test data (Kidney 5). Gold standard (red) shows the part of the whole kidney that was fully labeled and was part of the original competition dataset.

bioRxiv preprint doi: https://doi.org/10.1101/2024.08.25.609595; this version posted August 26, 2024. The copyright holder for this preprint (which was not certified by peer review) is the author/funder, who has granted bioRxiv a license to display the preprint in perpetuity. It is made available under aCC-BY 4.0 International license.

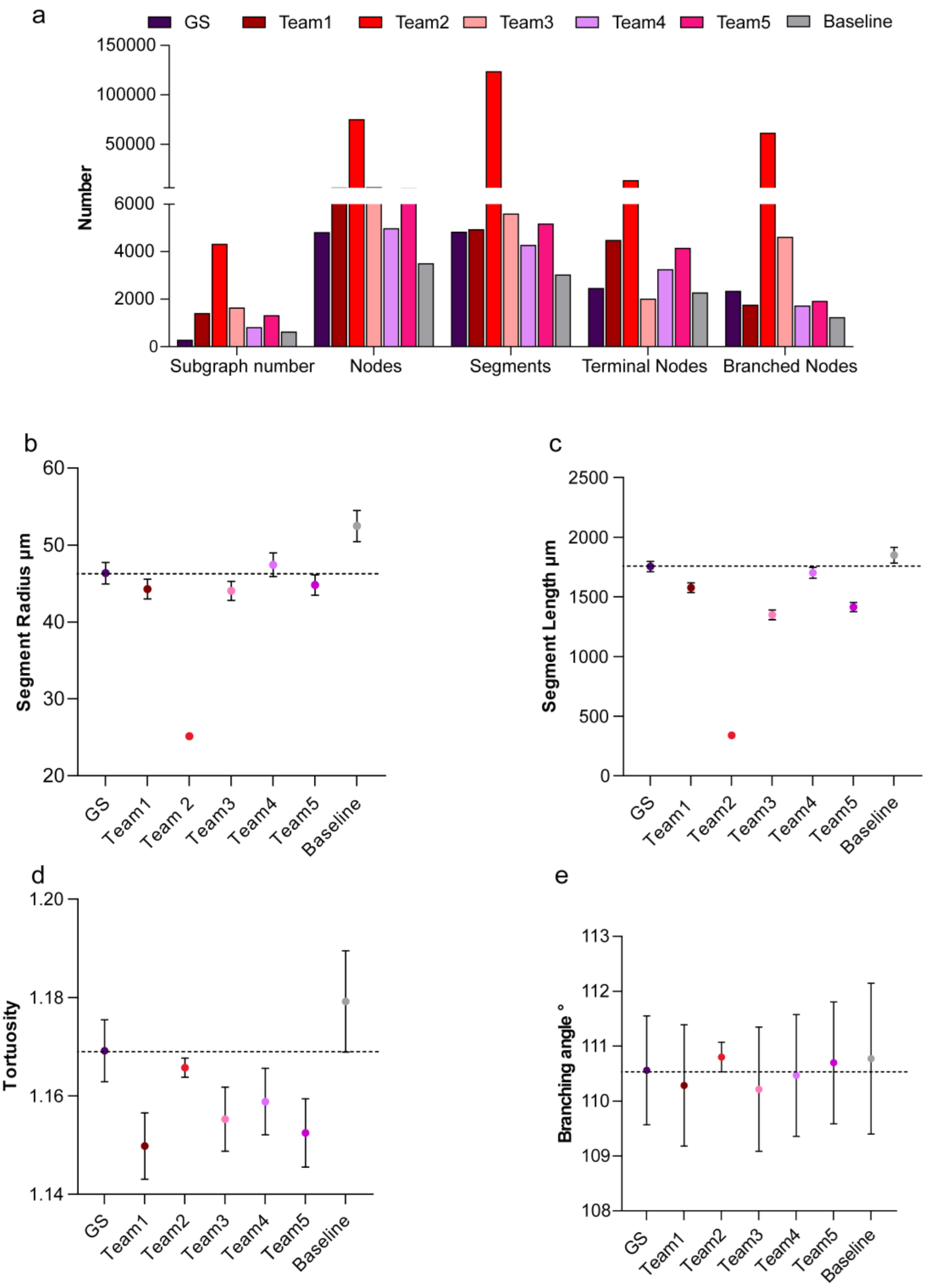

Supplementary Figure 5. a. Bar chart showing the number of subgraphs, nodes, segments, terminal nodes, and branched nodes for all team solutions as well as the baseline model predictions and gold standard (GS) labels for public test data. **b-e.** Plots showing the radius, length, tortuosity of segments, and the branching angle between segments; mean and 95% Confidence Interval (CI) are shown for all metrics.

bioRxiv preprint doi: https://doi.org/10.1101/2024.08.25.609595; this version posted August 26, 2024. The copyright holder for this preprint (which was not certified by peer review) is the author/funder, who has granted bioRxiv a license to display the preprint in perpetuity. It is made available under aCC-BY 4.0 International license.

Supplementary Tables

Supplementary Table 1. Listing of DOI and link for each dataset in the competition data.

Kaggle identifier	Donor identifier	Dataset name	DOI	Visualization link
**1**	LADAF-2021-17	LADAF-2021-17_kidney_right_complete-organ_25.0um_bm05	10.15151/ESRF-DC-1773966439	https://human-organ-atlas.esrf.eu/datasets/1773966586
**1**	LADAF-2021-17	LADAF-2021-17_kidney_right_VOI-03.1_2.6um_bm05	TBC	link to visualization
**2**	S-20-28	S-20-28_kidney_complete-organ_25.0um_bm05	TBC	link to visualization
**3**	LADAF-2020-27	LADAF-2020-27_kidney_left_complete-organ_25.08um_bm05	10.15151/ESRF-DC-572182553	https://human-organ-atlas.esrf.eu/datasets/572182201
**5**	LADAF-2021-17	LADAF-2021-17_kidney_left_complete-organ_25.14um_bm05	10.15151/ESRF-DC-1773965419	https://human-organ-atlas.esrf.eu/datasets/1773965003
**6**	LADAF-2022-13	LADAF-2022-13_kidney_2_complete-organ_15.77um_bm18	TBC	TBC

Supplementary Table 2. Scores for competition metric for top-5 teams and baseline model, including a brief summary.

Team	Summary of the model	Additional notes	Number of entries	Public score	Private score
1	Ensemble of two 2.5D convnext tiny U-net with	Data augmentation	278	0.895	0.774

bioRxiv preprint doi: https://doi.org/10.1101/2024.08.25.609595; this version posted August 26, 2024. The copyright holder for this preprint (which was not certified by peer review) is the author/funder, who has granted bioRxiv a license to display the preprint in perpetuity. It is made available under aCC-BY 4.0 International license.

	3 channels and custom loss	using random rotation			
2	3D UNet with binary focal loss	Data augmentation using random rotation and post-processing to remove unconnected vessels	33	0.043	0.755
3	UNet with MaxViT-Large 512 backbone	Creating pseudo labels from dense labels (train on kidney 1 and kidney 3 (dense labels) to generate supplemental labels for kidney 3 sparse. Repeat label generation (step 2) for kidney 2 sparse	114	0.846	0.727
4	Ensemble of 2D and 3D UNet with d4 TTA using boundary DoU loss	Creating pseudo labels (an ensemble of 2d models trained with the same setup but without CutMix to create pseudo labels from additional data 50um_LADAF-2020-31)	251	0.884	0.712
5	Ensemble of 3 UNet models (effnet_v2_s, maxvit_base and dpn68) with custom loss (CE + Dice + Focal)	Creating pseudo labels (same models trained to create pseudo labels from additional data from human organ atlas - LADAF-2020-31 kidney and LADAF-2020-27 spleen)	108	0.855	0.691
Baseline	NN-UNet Model	Default parameters	N/A	0.805	0.438

bioRxiv preprint doi: https://doi.org/10.1101/2024.08.25.609595; this version posted August 26, 2024. The copyright holder for this preprint (which was not certified by peer review) is the author/funder, who has granted bioRxiv a license to display the preprint in perpetuity. It is made available under aCC-BY 4.0 International license.

Supplementary Table 3. All metric scores for top-50 teams, on both test sets. Team ID is based on ranking on the final private leaderboard. Lower is better for ASSD scores. Other metrics are bounded between 0 and 1, higher values are better.

Team ID	Dataset ID	NSD (t=0)	NSD (t=1)	clDice	ASSD	Dice
1	kidney_5	0.8951	0.9569	0.833	1.3132	0.9283
1	kidney_6	0.7741	0.9252	0.7859	0.7877	0.8289
2	kidney_5	0.0431	0.0855	0.0134	40.5078	0.0119
2	kidney_6	0.756	0.8479	0.7991	2.7445	0.6758
3	kidney_5	0.8327	0.8959	0.7493	10.8552	0.8971
3	kidney_6	0.7279	0.8844	0.7428	1.0919	0.8174
4	kidney_5	0.8849	0.9423	0.8457	1.8925	0.9296
4	kidney_6	0.7122	0.8623	0.7342	1.4183	0.8144
5	kidney_5	0.8855	0.9554	0.8279	1.1549	0.9279
5	kidney_6	0.6918	0.8612	0.7088	1.5988	0.8041
6	kidney_5	0.8862	0.9477	0.8411	0.7425	0.9315
6	kidney_6	0.6819	0.8055	0.6659	1.8331	0.792
7	kidney_5	0.8594	0.9156	0.7959	6.1061	0.9152
7	kidney_6	0.6768	0.8492	0.7139	1.4423	0.7802
8	kidney_5	0.879	0.9431	0.8088	1.4752	0.9275
8	kidney_6	0.6753	0.8585	0.7015	1.4384	0.778
9	kidney_5	0.8272	0.8998	0.7227	4.2324	0.9088
9	kidney_6	0.6651	0.8843	0.7343	1.1351	0.7758
10	kidney_5	0.8103	0.8795	0.7159	10.2946	0.8912
10	kidney_6	0.6572	0.8496	0.699	1.4754	0.7781
11	kidney_5	0.6132	0.7349	0.6529	17.6852	0.649
11	kidney_6	0.6559	0.7625	0.6824	3.2546	0.6951
12	kidney_5	0.6362	0.8144	0.5687	7.2457	0.801
12	kidney_6	0.6488	0.8012	0.6436	3.2204	0.7075
13	kidney_5	0.8581	0.9165	0.7899	3.1011	0.9138

bioRxiv preprint doi: https://doi.org/10.1101/2024.08.25.609595; this version posted August 26, 2024. The copyright holder for this preprint (which was not certified by peer review) is the author/funder, who has granted bioRxiv a license to display the preprint in perpetuity. It is made available under aCC-BY 4.0 International license.

13	kidney_6	0.6479	0.8382	0.6593	1.3991	0.7674
14	kidney_5	0	0		inf	0
14	kidney_6	0.6462	0.8177	0.6888	2.1497	0.7571
15	kidney_5	0.8228	0.9023	0.7518	4.6402	0.9011
15	kidney_6	0.6359	0.8184	0.6718	1.8454	0.7831
16	kidney_5	0.8887	0.9503	0.8065	1.8009	0.9285
16	kidney_6	0.6347	0.8635	0.6904	1.2871	0.7635
17	kidney_5	0.8137	0.8884	0.6991	2.1368	0.9077
17	kidney_6	0.6345	0.823	0.6739	1.8226	0.7782
18	kidney_5	0.8368	0.8961	0.7846	7.3325	0.8789
18	kidney_6	0.6245	0.8353	0.7032	5.0582	0.736
19	kidney_5	0.8133	0.8869	0.6914	1.3084	0.9074
19	kidney_6	0.6214	0.7885	0.6391	4.6257	0.724
20	kidney_5	0.8568	0.9287	0.7776	2.5528	0.9155
20	kidney_6	0.6167	0.8465	0.7333	1.4698	0.6847
21	kidney_5	0.8199	0.891	0.7248	7.8449	0.896
21	kidney_6	0.6141	0.8227	0.6838	2.1755	0.7271
22	kidney_5	0.8722	0.943	0.802	1.5679	0.921
22	kidney_6	0.6117	0.8155	0.6539	2.3056	0.7248
23	kidney_5	0.8507	0.9086	0.7707	1.6365	0.9179
23	kidney_6	0.6103	0.7729	0.654	3.0569	0.7025
24	kidney_5	0.8735	0.9414	0.831	2.6179	0.9188
24	kidney_6	0.6088	0.8202	0.6746	4.1682	0.7267
25	kidney_5	0	0		inf	0
25	kidney_6	0.608	0.8119	0.6585	1.9342	0.7667
26	kidney_5	0.8676	0.9379	0.7936	1.3814	0.9224
26	kidney_6	0.606	0.7712	0.6188	3.2132	0.7469
27	kidney_5	0.8652	0.924	0.8019	5.6499	0.9108
27	kidney_6	0.6035	0.7922	0.6504	2.6637	0.738

bioRxiv preprint doi: https://doi.org/10.1101/2024.08.25.609595; this version posted August 26, 2024. The copyright holder for this preprint (which was not certified by peer review) is the author/funder, who has granted bioRxiv a license to display the preprint in perpetuity. It is made available under aCC-BY 4.0 International license.

28	kidney_5	0.8496	0.9096	0.77	5.6527	0.9124
28	kidney_6	0.602	0.8053	0.6509	3.3284	0.7603
29	kidney_5	0.8594	0.9187	0.7875	1.2754	0.9178
29	kidney_6	0.5992	0.7264	0.594	3.1979	0.6882
30	kidney_5	0.8703	0.9338	0.805	2.6242	0.9242
30	kidney_6	0.5988	0.7907	0.6391	2.0989	0.7483
31	kidney_5	0.8301	0.9013	0.7501	2.9544	0.9069
31	kidney_6	0.5982	0.7992	0.6869	3.4409	0.7232
32	kidney_5	0.8561	0.9326	0.7795	1.5592	0.9157
32	kidney_6	0.5968	0.796	0.6446	2.1674	0.7349
33	kidney_5	0.8432	0.9163	0.7432	3.3218	0.9129
33	kidney_6	0.5959	0.7973	0.6252	6.5184	0.745
34	kidney_5	0.8754	0.9397	0.8121	1.9118	0.9235
34	kidney_6	0.5944	0.8322	0.6843	1.6091	0.7481
35	kidney_5	0.8503	0.9269	0.764	2.2443	0.9123
35	kidney_6	0.5943	0.7931	0.6531	3.0259	0.6511
36	kidney_5	0.8952	0.956	0.8509	0.7754	0.9322
36	kidney_6	0.5936	0.7696	0.609	2.7045	0.7683
37	kidney_5	0.6082	0.7485	0.5749	24.0377	0.7656
37	kidney_6	0.5905	0.7227	0.5769	6.9668	0.7079
38	kidney_5	0.6804	0.7684	0.6423	19.6523	0.7369
38	kidney_6	0.5905	0.7317	0.6036	5.5285	0.6497
39	kidney_5	0	0		inf	0
39	kidney_6	0.5896	0.8272	0.6714	1.733	0.7546
40	kidney_5	0.7982	0.8653	0.7004	11.4582	0.8858
40	kidney_6	0.5875	0.8007	0.639	2.9754	0.7554
41	kidney_5	0.8348	0.9147	0.7845	4.2633	0.9016
41	kidney_6	0.587	0.78	0.6293	4.6619	0.7572
42	kidney_5	0.8811	0.9477	0.815	0.8389	0.9261

bioRxiv preprint doi: https://doi.org/10.1101/2024.08.25.609595; this version posted August 26, 2024. The copyright holder for this preprint (which was not certified by peer review) is the author/funder, who has granted bioRxiv a license to display the preprint in perpetuity. It is made available under aCC-BY 4.0 International license.

42	kidney_6	0.5862	0.7822	0.6411	5.1143	0.6628
43	kidney_5	0.8711	0.9382	0.7985	1.7559	0.9229
43	kidney_6	0.5848	0.8085	0.6718	2.0455	0.6903
44	kidney_5	0.8761	0.9428	0.8073	1.9085	0.9241
44	kidney_6	0.5848	0.8102	0.6644	4.4716	0.6803
45	kidney_5	0.8941	0.9541	0.8403	0.7352	0.9297
45	kidney_6	0.584	0.827	0.6803	3.0314	0.6334
46	kidney_5	0.8065	0.8823	0.6973	2.8374	0.9005
46	kidney_6	0.5829	0.7626	0.6044	2.5261	0.7397
47	kidney_5	0.8283	0.9073	0.7383	3.4346	0.905
47	kidney_6	0.5824	0.7888	0.6594	2.2022	0.7001
48	kidney_5	0.8741	0.942	0.8172	0.8616	0.9241
48	kidney_6	0.5809	0.7819	0.6335	7.6572	0.7435
49	kidney_5	0.7685	0.8545	0.715	12.7754	0.8565
49	kidney_6	0.5788	0.738	0.6031	3.248	0.7292
50	kidney_5	0.8364	0.9029	0.7445	3.5469	0.9101
50	kidney_6	0.5782	0.799	0.6574	2.2052	0.7254
